# Identification of biomarker‐by‐treatment interactions in randomized clinical trials with survival outcomes and high‐dimensional spaces

**DOI:** 10.1002/bimj.201500234

**Published:** 2016-11-15

**Authors:** Nils Ternès, Federico Rotolo, Georg Heinze, Stefan Michiels

**Affiliations:** ^1^ INSERM U1018, CESP Université Paris‐Sud, Université Paris‐Saclay Villejuif F‐94805 France; ^2^ Gustave Roussy, Paris‐Saclay Service de Biostatistique et d'Epidémiologie Villejuif F‐94805 France; ^3^ Section for Clinical Biometrics, Center for Medical Statistics, Informatics and Intelligent Systems Medical University of Vienna Vienna A‐1090 Austria

**Keywords:** Biomarker‐by‐treatment interactions, High‐dimensional, Precision medicine, Stratified medicine, Survival, Variable selection

## Abstract

Stratified medicine seeks to identify biomarkers or parsimonious gene signatures distinguishing patients that will benefit most from a targeted treatment. We evaluated 12 approaches in high‐dimensional Cox models in randomized clinical trials: penalization of the biomarker main effects and biomarker‐by‐treatment interactions (full‐lasso, three kinds of adaptive lasso, ridge+lasso and group‐lasso); dimensionality reduction of the main effect matrix via linear combinations (PCA+lasso (where PCA is principal components analysis) or PLS+lasso (where PLS is partial least squares)); penalization of modified covariates or of the arm‐specific biomarker effects (two‐I model); gradient boosting; and univariate approach with control of multiple testing. We compared these methods via simulations, evaluating their selection abilities in null and alternative scenarios. We varied the number of biomarkers, of nonnull main effects and true biomarker‐by‐treatment interactions. We also proposed a novel measure evaluating the interaction strength of the developed gene signatures. In the null scenarios, the group‐lasso, two‐I model, and gradient boosting performed poorly in the presence of nonnull main effects, and performed well in alternative scenarios with also high interaction strength. The adaptive lasso with grouped weights was too conservative. The modified covariates, PCA+lasso, PLS+lasso, and ridge+lasso performed moderately. The full‐lasso and adaptive lassos performed well, with the exception of the full‐lasso in the presence of only nonnull main effects. The univariate approach performed poorly in alternative scenarios. We also illustrate the methods using gene expression data from 614 breast cancer patients treated with adjuvant chemotherapy.

## 1 Introduction

In randomized controlled trials (RCT), the treatment benefit is often measured as an average across the study population but, in the era of stratified medicine, an increasing interest is devoted to identify patients more likely to benefit from the treatment. Unaccounted biomarker‐by‐treatment interactions can dramatically lower the statistical power (Betensky et al., 2002), which may be one of the reasons why RCTs have often failed to show a benefit of the drug in oncology (Buyse and Michiels, 2013). Hence, it is important to identify in RCTs (prospectively or retrospectively) these biomarker‐by‐treatment interactions, also called treatment‐effect modifiers or predictive biomarkers (Michiels et al., 2007; Royston and Sauerbrei, 2008; Buyse et al., 2013). An example is the phase III IPASS trial that showed an overall benefit on progression‐free survival (PFS) of gefitinib as compared to carboplatin plus paclitaxel in advanced nonsmall cell lung cancer patients (Mok et al., 2009), with a high benefit in patients with epidermal growth factor receptor (EGFR) mutation and a harm in patients without it. Another treatment‐effect modifier is the Kirsten rat sarcoma (KRAS) mutation for the effect on PFS of anti‐EGFR monoclonal antibodies in advanced colorectal cancer (Amado et al., 2008). Recently, also gene signatures (i.e., combination of multiple treatment‐effect modifiers) have been proposed: for example, an 8‐gene and a 14‐gene signature for trastuzumab in early breast cancer (Pogue‐Geile et al., 2013; Perez et al., 2015) and a 84‐gene signature for MAGE‐A3 immunotherapy in melanoma and nonsmall cell lung cancer (Ulloa‐Montoya et al., 2013).

From a statistical viewpoint, Rothwell (2005) put forward that the only reliable approach for assessing the predictiveness of biomarkers is to test their interaction with the treatment. Thus, the general framework for identifying treatment‐effect modifiers is a model with the main effects of both the treatment and the biomarkers, and the biomarker‐by‐treatment interactions. However, with genomic biomarkers, high dimensionality (much more biomarkers *p* than sample size *n*) often makes the model nonidentifiable. We propose several methods to select a sparse set of treatment‐effect modifiers among a large number of candidates in an RCT in the framework of Cox regression models (Cox, 1972). In Section 2, we describe 12 possible approaches. In Section 3, we evaluate via simulations their selection performance and we consider a novel measure to evaluate the biomarker‐by‐treatment interaction strength of the developed gene signatures. In Section 4, we present an analysis of publicly available gene expression data in breast cancer. In Section 5, we discuss the findings.

## 2 Methods

### 2.1 Penalties on the full biomarker‐by‐treatment interaction model

In a proportional hazards regression model, the full biomarker‐by‐treatment interaction model is

(1) $$h\left(t|T,X\right)=h_{0}\left(t\right)\exp\left(\alpha T+\;\sum_{i\;=\;1}^{p}\beta_{i}X_{i}+\;\sum_{i\;=\;1}^{p}\gamma_{i}X_{i}T\right)$$

withα, β, and γ the regression coefficients for the treatment *T* (+0.5 the experimental and −0.5 the control arm), the standardized biomarkers $X_{i}$, *i* = 1, …, *p*, and their interactions $X_{i}T$, respectively. Of note, the second sum in (1) is the component estimating the biomarker‐dependent treatment effect. Semiparametric estimates (Cox, 1972) of the regression parameters are obtained by maximizing the partial log‐likelihood l(β). However, in a high‐dimensional setting (2*p* + 1≫n), the model is nonidentifiable. To overcome this issue, penalized regression maximizes the penalized log‐likelihood l(β,γ)−p(λ,β,γ), by adding a penalty p(λ,β,γ) such as the ridge (Hoerl and Kennard, 1970) or the lasso (Tibshirani, 1996, 1997). In all cases that we consider here, penalization is applied to ($\beta_{i},\gamma_{i})$ whereas α remains unpenalized.

The first penalization considered uses the lasso penalty for both the$\;\beta_{i}$’s and the$\;\gamma_{i}$’s to perform variable selection and hence, identify treatment‐effect modifiers:

$$p\left(\lambda ,\beta ,\gamma \right)=\;\lambda \left(\sum_{i\;=\;1}^{p}\left|\beta_{i}\right|+\sum_{i\;=\;1}^{p}\left|\gamma_{i}\right|\right).$$

The main effects and the interactions are equally penalized (same shrinkage parameterλ). We call this approach **full‐lasso** throughout the paper. This approach lacks the hierarchy constraint: the main effect of a biomarker can be discarded ($\beta_{i}=\;0)$ irrespective of whether the associated interaction $\gamma_{i}$ is. Although this can affect the interpretability of $\gamma_{i}$ and the calibration of the model (Bien et al., 2013), this is of minor importance in the context of selection.

Despite the simplicity of this method, main effects and interactions can have very different sizes. It seems more appropriate to penalize them unequally: using differently weighted penalties for the $\beta_{i}$’s and the $\gamma_{i}$’s, the approach is similar to the adaptive lasso (alasso), which penalizes large coefficients less than smaller ones to stress differences between them (Zou, 2006; Zhang and Lu, 2007). In this spirit, we estimate weights in a preliminary model including the treatment and all the biomarker main effects $\beta_{Ri}$ and interactions with the treatment$\;\gamma_{Ri}$, with a ridge penalty on the$\;\beta_{Ri}$’s and the$\;\gamma_{Ri}$’s

$$\lambda_{2}\left(\sum_{i\;=\;1}^{p}\beta_{Ri}^{2}+\sum_{i\;=\;1}^{p}\gamma_{Ri}^{2}\right)$$

to control their magnitude. In a second stage, two strategies to estimate the weights are considered: one with biomarker‐specific (**alasso (Sw)**) and one with grouped (**alasso (Gw)**) weights. The alasso (Sw) strategy estimates weights as the inverse of the absolute ridge coefficients:

$$p\left(\lambda ,\beta ,\gamma \right)=\;\lambda \left(\sum_{i\;=\;1}^{p}\frac{1}{\left|\beta_{Ri}\right|}\left|\beta_{i}\right|+\sum_{i\;=\;1}^{p}\frac{1}{\left|\gamma_{Ri}\right|}\left|\gamma_{i}\right|\right).$$

The alasso (Gw) strategy estimates a common weight for all$\;\beta_{i}$’s and one for all$\;\gamma_{i}$’s as their average:

$$p\left(\lambda ,\beta ,\gamma \right)=\;\lambda \left(\frac{1}{\beta_{R}}\sum_{i\;=\;1}^{p}\left|\beta_{i}\right|+\frac{1}{\gamma_{R}}\sum_{i\;=\;1}^{p}\left|\gamma_{i}\right|\right),\;\;\beta_{R}=\frac{1}{p}\;\sum_{i\;=\;1}^{p}\left|\beta_{Ri}\right|,\;\;\gamma_{R}=\frac{1}{p}\;\sum_{i\;=\;1}^{p}\left|\gamma_{Ri}\right|.$$

In order to force the hierarchy constraint, we considered a further approach (**ridge+lasso**) with a ridge penalty on the main effects, which are then all kept in the model while controlling for overfitting:

$$p\left(\lambda ,\lambda_{2},\beta ,\gamma \right)=\;\lambda_{2}\sum_{i\;=\;1}^{p}\beta_{i}^{2}+\;\lambda \sum_{j\;=\;1}^{p}\left|\gamma_{i}\right|$$

Because optimization of both λ and$\;\lambda_{2}$ is computationally demanding and weakens generalizability, we favor precise optimization of λ with rough selection of$\;\lambda_{2}$. In the context of the elastic net, Zou and Hastie (2005) estimated λ for a grid of given$\;\lambda_{2}$ and chose the best ($\lambda ,\lambda_{2}$). In our case, we first estimate the$\;\beta_{i}$’s in a model without interactions and then fixed them via an offset in the final model (1). Sensitivity analyses showed no relevant difference between the two strategies (data not shown).

To the aim of forcing the hierarchy constraint while performing selection on both the $\beta_{i}$’s and the$\;\gamma_{i}$, we also consider a **group‐lasso** approach (Yuan and Lin, 2006), which selects prespecified groups of variables. In our context, *p* groups $\left(\beta_{i},\;\gamma_{i}\right)$ are defined:

$$p\left(\lambda ,\beta ,\gamma \right)=\;\lambda \;\sum_{i\;=\;1}^{p}\sqrt{\beta_{i}^{2}+\gamma_{i}^{2}}.$$

### 2.2 The modified covariates approach

Rather than the full interaction model (1), Tian et al. (2014) proposed a model with no main effects and **modified covariates**, defined as the product of each $X_{i}$ and the treatment *T* (= ±0.5):

(2) $$h\left(t|T,X\right)=h_{0}\left(t\right)\exp\left(\sum_{i\;=\;1}^{p}\gamma_{i}M_{i}\right),\;\;\;M_{i}=X_{i}\;T.$$

This corresponds to model (1) with only the interaction part and no main effects. The lasso penalty is used to perform variable selection in model (2).

### 2.3 Dimension reduction of the biomarker main effects

A compromise between including only interactions and all the main effects is achieved by reducing the space dimension of the main effects through a principal components analysis (**PCA**, Hastie et al., 2001). However, the principal components are computed independently of the outcome. To overcome this drawback, partial least squares (**PLS**, Martens and Naes, 1989) regression computes the linear combinations that are the most associated to survival. These orthogonal transformations allow reducing the number of parameters in the model:

(3) $$h\left(t|T,Z,X\right)=h_{0}\left(t\right)\exp\left(\alpha T+\;\sum_{k\;=\;1}^{K}\beta_{k}Z_{k}+\;\sum_{i\;=\;1}^{p}\gamma_{i}X_{i}T\right),$$

with $Z_{1},\ldots ,Z_{K}$ the first K≪p linear combinations of the biomarkers. We apply a lasso penalty to the interactions for identifying treatment‐effect modifiers and αremains unpenalized.

### 2.4 The gradient boosting

Boosting techniques exploit the repeated fitting of a weak estimator to iteratively obtain a model maximizing the partial log‐likelihood. In a high‐dimensional setting, the process starts from the null model and one coefficient is updated at each step. This iterative process stops when the model achieves a balance between bias and variance. In this study, we focus on gradient boosting (Friedman, 2001; Bühlmann and Yu, 2003) which consists in a componentwise L2‐boosting: the possible updates are computed on each predictor, and the best is retained after penalization to update the model. The treatment effect is estimated preliminarily and then fixed as an offset. The gradient boosting does not impose the hierarchy constraint.

### 2.5 Univariate approach with FDR control

A straightforward approach consists in testing the interaction of the treatment with each biomarker through a Wald test in a model incorporating the treatment, the biomarker and their interaction (Michiels et al., 2011). In order to perform variable selection, we adjust the interaction *p*‐values using the Benjamini and Hochberg procedure (1995) and keep biomarkers with adjusted *p*‐value < 0.05.

### 2.6 Arm‐specific prognostic effects

The last two approaches considered estimate the arm‐specific prognostic effects of biomarkers and then compare them to identify treatment‐effect modifiers. The so‐called two‐interaction model (**two‐I model**) replaces in model (1) the main effects by a second interaction term for the control arm:

(4) $$h\left(t|T,X\right)=h_{0}\left(t\right)\exp\left(\alpha T+\;\sum_{i\;=\;1}^{p}\left(\gamma_{i+}X_{i}I\left(T\;=\;0.5\right)+\gamma_{i-}X_{i}I\left(T\;=\;-0.5\right)\right)\right),$$

$\gamma_{i+}$ and $\gamma_{i-}$ representing the prognostic effect of the *i*‐th biomarker in the experimental (T=0.5) and control (T=−0.5) arm, respectively. A lasso penalty is applied to both the$\;\gamma_{i+}$’s and the$\;\gamma_{i-}$’s. A biomarker *i* is considered as treatment‐effect modifier only if one of the two effects$\;(\gamma_{i+}$, $\gamma_{i-})$ is kept in the model and the other is not.

Alternatively, we incorporate the estimates of the two‐interaction model (4) in the full interaction model (1) as weights of an adaptive lasso penalty. Let $\gamma_{Ri+}$ and $\gamma_{Ri-}$ be the estimates of model (4) under a ridge penalty; model (1) is now penalized as follows:

$$p\left(\lambda ,\beta ,\gamma \right)=\;\lambda \left(\sum_{i\;=\;1}^{p}\frac{1}{\left|\gamma_{Ri+}+\gamma_{Ri-}\right|+\left|\gamma_{Ri+}-\gamma_{Ri-}\right|}\left|\beta_{i}\right|+\sum_{i\;=\;1}^{p}\frac{1}{\left|\gamma_{Ri+}-\gamma_{Ri-}\right|}\left|\gamma_{i}\right|\right).$$

These arm‐specific prognostic weights (**Aspw**) yield smaller weights for interactions with larger difference between the two arm‐specific prognostic effects. In addition, the higher such difference, the lower the weight of the associated main effect, which favors (even though not forces) the hierarchy constraint.

### 2.7 Implementation

The methods outlined so far were implemented in R v3.1.2 with packages: glmnet (Friedman et al., 2010; Friedman et al., 2016) for lasso and ridge penalties, grplasso (Meier, 2015) for group‐lasso penalty, corpcor (Schäfer et al., 2015) for principal components, plsRcox (Bertrand et al., 2014) for PLS, and mboost (Hothorn et al., 2016) for gradient boosting. Of note, the grplasso R‐package can only be used for generalized linear models, thus, we implemented a Poisson model over two‐month intervals, corresponding to a piecewise constant hazard model which approximates rather well the Breslow estimator in the Cox model (Pawitan, 2005). We use fivefold cross**‐**validation (CV, Verweij and van Houwelingen, 1993, 1994) to estimate the shrinkage parameters λ and λ_2_, the number of principal components *K*, and the number of iterations for the gradient boosting (with shrinkage factor (ν) fixed to 0.1). As CV for the PLS gave extremely poor results (data not shown), we decided to keep only the first component. The R code is provided in the supplementary material.

## 3 Simulation study

We performed simulations to compare methods in terms of selection of the treatment‐effect modifiers in high‐dimensional Cox models. We also evaluated the interaction strength of the selected biomarkers.

### 3.1 Data generation

We generated *p* = 500 or 1000 unit‐variance ($\sigma^{2}=\;1)$ Gaussian biomarkers with autoregressive correlation ($\sigma_{ij}=0.7^{\left|i-j\right|}$) within 25‐ or 50‐biomarker blocks, respectively. A total of *n* = 500 patients per data set were randomly assigned (1:1) to the experimental or control arm. We generated exponential survival times by fixing for each treatment arm:$\;m_{0}$, the baseline (i.e., $\;x^{T}={\left(0,\;\ldots ,\;0\right)}^{T}$) median survival time; *m*_1_, the median survival time for a one‐unit increase of an active biomarker; and the associated log‐hazard ratio $\beta \;=\log(m_{0}/m_{1})$. We generated independent censoring from a U(2,5) distribution, reflecting a trial with three‐year accrual and two‐year follow‐up.

### 3.2 Simulation scenarios

Table 1 shows the null (i.e., no treatment‐effect modifier) and alternative scenarios (i.e., at least one treatment‐effect modifier) considered in the simulations. In the complete null scenario, $m_{0}=m_{1}\;=\;1$, whatever the treatment. In the second null scenario, there was a strong treatment effect and in the third, 10 (for *p* = 500) or 20 (for *p* = 1000) biomarkers were strongly prognostic. In alternative scenarios 4 and 5, no biomarker had an effect in the control arm, while one and 10 or 20 treatment‐effect modifiers doubled the median survival time in the treatment arm (HR+ = 0.5). In scenario 6, we combined prognostic biomarkers (HR+ = HR– = 0.5) and treatment‐effect modifiers (HR– = 1, HR+ = 0.5). Simulations were performed with *p* = 500 (scenarios 1a–6a) and *p* = 1000 (scenarios 1b–6b). The censoring rate was 10–37%. We also considered a setting tuned on the application of Section 4 (scenario 1c–6c): higher censoring (60–80%) and lower biomarker effects (randomly drawn from U(−0.5,−0.1) for main effects and U(−0.7,−0.1) for interactions). The biomarkers were randomly allocated to correlation blocks, independently of their effects. As sensitivity analyses, we considered Weibull times with shape 0.5 and 2 (Supporting Information Fig. S1), and we forced correlation between predictive, and between predictive and prognostic biomarkers (Supporting Information Fig. S2). For each data set, another one with the same parameters was generated for external validation to evaluate the interaction strength of the developed signatures.

**Table 1:** Design of the simulation study

Two hundred fifty repetitions per scenario			Median survival time (years)		Hazard ratio		Average censoring probability	
			*X* = 0		*h*(*X* = 1)/*h*(*X* = 0)			
			*T^–^*	*T^+^*	*T^–^*	*T^+^*	*T^–^*	*T^+^*
*p* = 500 biomarkers	(1a)	Complete null	1.0	1.0	1.0	1.0	0.10	0.11
	(2a)	Treatment effect only	1.0	2.0	1.0	1.0	0.10	0.31
	(3a)	10 prognostic markers	1.0	1.0	0.5	0.5	0.30	0.30
	(4a)	One treatment modifier	1.0	1.0	1.0	0.5	0.10	0.15
	(5a)	10 treatment modifiers	1.0	1.0	1.0	0.5	0.10	0.29
	(6a)	10 treatment modifiers +	1.0	1.0	1.0	0.5	0.30	0.35
		10 prognostic markers			0.5	0.5		
*p* = 1000 biomarkers	(1b)	Complete null	1.0	1.0	1.0	1.0	0.11	0.10
	(2b)	Treatment effect only	1.0	2.0	1.0	1.0	0.11	0.31
	(3b)	20 prognostic markers	1.0	1.0	0.5	0.5	0.35	0.35
	(4b)	One treatment modifier	1.0	1.0	1.0	0.5	0.11	0.15
	(5b)	20 treatment modifiers	1.0	1.0	1.0	0.5	0.11	0.35
	(6b)	20 treatment modifiers +	1.0	1.0	1.0	0.5	0.35	0.39
		20 prognostic markers			0.5	0.5		
*p* = 500 biomarkers	(1c)	Complete null	1.0	1.0	1.0	1.0	0.65	0.66
	(2c)	Treatment effect only	1.0	2.0	1.0	1.0	0.65	0.81
	(3c)	10 prognostic markers	1.0	1.0	exp ($\beta_{m}$)	exp ($\beta_{m}$)	0.60	0.61
	(4c)	One treatment modifier	1.0	1.0	1.0	exp ($\beta_{i}$)	0.66	0.64
	(5c)	10 treatment modifiers	1.0	1.0	1.0	exp ($\beta_{i}$)	0.66	0.59
	(6c)	10 treatment modifiers +	1.0	1.0	1.0	exp ($\beta_{i}$)	0.61	0.58
		10 prognostic markers			exp ($\beta_{m}$)	exp ($\beta_{m}$)		

$T^{-}$: control arm, $T^{+}$: experimental arm, *X*: biomarker, $\beta_{m}$ randomly drawn from *U*(−0.5, −0.1), $\beta_{i}$ randomly drawn from *U*(−0.7, −0.1).

### 3.3 Evaluation criteria

The primary objective of this study was to compare methods to correctly identify the true treatment‐effect modifiers. The secondary objective was to evaluate how well the selected biomarkers predict a differential treatment effect for future patients.

We considered two criteria for selection of true positive (TP) treatment‐effect modifiers: the false discovery rate FDR = FP/(TP + FP), that is, the rate of false positive (FP) biomarkers among those selected (Genovese and Wasserman, 2002), and the false negative rate FNR = FN/(TP + FN), that is, the rate of false negatives (FNs) among the true treatment‐effect modifiers (Pawitan et al., 2013). In scenarios including main effects, we reported the number of prognostic FPs. We also computed the area under the precision‐recall curve (AUPRC, Davis and Goadrich, 2006), translating the ability of discarding inactive biomarkers more likely than active ones independently of the tuning parameters. The AUPRC, based on FDR and FNR, is more pertinent than the area under the ROC curve when there are many more inactive than active biomarkers.

Selection methods can also serve as a global test of the presence of any interaction signal in the biomarker set (Michiels et al., 2011; Michiels and Rotolo, 2015); the empirical rejection probability is the type‐I error rate in null scenarios (corresponding to the FDR as any selected biomarker is a FP), and is the power in the alternative scenarios.

The biomarker‐treatment score for patients in the validation set (V) is the cross product between the coefficients ${\hat{\gamma }}^{\mathrm{tr}}$ of the interactions retained in the training set (tr) and their biomarkers:

η^j=∑s∈Hγ^s tr ×Xj,sv with H={s∣γ^s tr ≠0}.

The lower the score, the higher the treatment benefit. We propose a gene signature interaction strength criterion similar to Schemper (1988) and Michiels et al. (2011), measuring the concordance between ${\hat{\eta }}_{j}$ and the survival time in each treatment arm. We estimated such within‐arm concordance via the C‐statistic of Uno, one of the least biased estimators in the presence of censoring (Uno et al., 2011). Then, we computed the difference of the two C‐statistics (ΔC‐statistics): the larger the difference, the higher the interaction strength. We computed the ΔC‐statistics both in the training and validation sets.

### 3.4 Results

**Figure 1:**
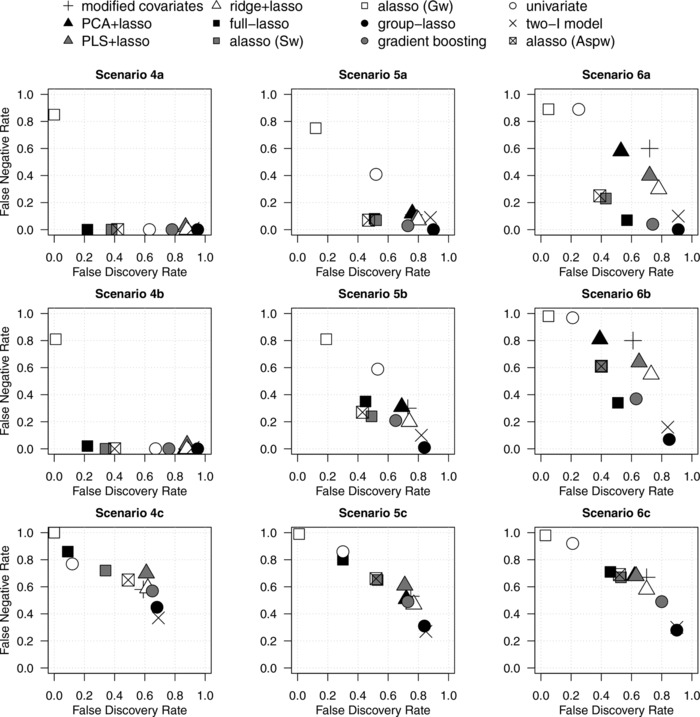
False Negative Rate (FNR) against the False Discovery Rate (FDR) in alternative scenarios. Average quantities across 250 replications.

**Figure 2:**
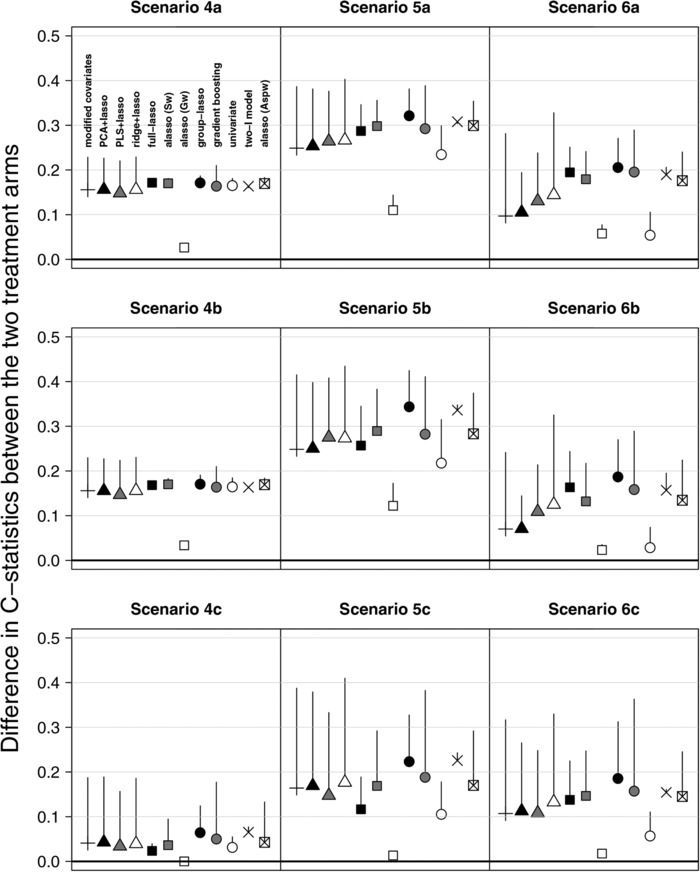
Difference in arm‐specific C‐statistics (ΔC‐statistics) in alternative scenarios in the training and validation set. Vertical lines represent the reduction in ΔC‐statistic from the training set to the validation set. Average quantities across 250 replications.

Tables 2 and 3 and Figs. 1 and 2 summarize the results of simulations. The methods can be grouped into four main groups according to their performances.

**Table 2:** Proportion of models selecting at least one biomarker‐by‐treatment interaction for all the methods among 250 replications

		Univariate	Modified covariates	PCA+lasso	PLS+lasso	Ridge+lasso	Group‐lasso	Two‐I model	Full‐lasso	Alasso (sw)	Alasso (gw)	Alasso (aspw)	Gradient boosting
Null scenarios	Scenario 1a	0.07	0.39	0.38	0.36	0.39	0.48	0.41	0.01	0.14	0.00	0.42	0.68
	Scenario 2a	0.06	0.35	0.43	0.38	0.39	0.56	0.44	0.01	0.12	0.00	0.37	0.66
	Scenario 3a	0.06	0.37	0.24	0.41	0.47	1.00	1.00	0.88	0.20	0.00	0.32	1.00
	Scenario 1b	0.06	0.38	0.35	0.32	0.38	0.52	0.40	0.01	0.12	0.00	0.36	0.68
	Scenario 2b	0.04	0.41	0.43	0.43	0.38	0.52	0.38	0.02	0.16	0.00	0.38	0.69
	Scenario 3b	0.08	0.45	0.27	0.42	0.58	1.00	1.00	0.98	0.32	0.00	0.55	1.00
	Scenario 1c	0.05	0.56	0.57	0.50	0.56	0.56	0.40	0.03	0.25	0.00	0.51	0.73
	Scenario 2c	0.04	0.55	0.56	0.61	0.56	0.46	0.37	0.00	0.13	0.00	0.44	0.65
	Scenario 3c	0.06	0.53	0.49	0.58	0.60	1.00	1.00	0.51	0.63	0.00	0.73	1.00
Alternative scenarios	Scenario 4a	1.00	1.00	1.00	0.99	1.00	1.00	1.00	1.00	1.00	0.15	1.00	1.00
	Scenario 5a	0.99	1.00	1.00	1.00	1.00	1.00	1.00	1.00	1.00	0.66	1.00	1.00
	Scenario 6a	0.59	0.90	0.80	0.94	0.99	1.00	1.00	1.00	1.00	0.55	1.00	1.00
	Scenario 4b	1.00	1.00	1.00	0.99	1.00	1.00	1.00	0.98	1.00	0.19	1.00	1.00
	Scenario 5b	1.00	1.00	0.98	1.00	1.00	1.00	1.00	0.98	1.00	0.78	1.00	1.00
	Scenario 6b	0.43	0.78	0.64	0.90	0.98	1.00	1.00	1.00	1.00	0.28	1.00	1.00
	Scenario 4c	0.27	0.66	0.70	0.65	0.68	0.72	0.76	0.20	0.48	0.00	0.65	0.76
	Scenario 5c	0.74	0.94	0.93	0.89	0.98	0.99	1.00	0.73	0.96	0.10	0.97	0.99
	Scenario 6c	0.51	0.86	0.83	0.82	0.91	1.00	1.00	0.97	0.99	0.14	0.99	1.00

Null scenarios: type‐I error or FDR. Alternative scenarios: power.

**Table 3:** Selection performance of the methods in alternative scenarios

		Univariate	Modified covariates	PCA+lasso	PLS+lasso	Ridge+lasso	Group‐lasso	Two‐I model	Full‐lasso	Alasso (sw)	Alasso (gw)	Alasso (aspw)	Gradient boosting
Scenario 4a	Selected biomarkers	4	14	13	14	14	24	18	2	2	0	3	7
	TP / FP(pFP)	1 / 3(0)	1 / 13(0)	1 / 12(0)	1 / 13(0)	1 / 13(0)	1 / 23(0)	1 / 18(0)	1 / 1(0)	1 / 1(0)	0 / 0(0)	1 / 2(0)	1 / 6(0)
	AUPRC	1.00	0.98	0.98	0.95	0.98	0.99		0.99	0.99	0.99	0.99	0.99
Scenario 5a	Selected biomarkers	14	43	42	49	49	100	78	20	20	3	18	37
	TP / FP (pFP)	6 / 8(0)	9 / 34(0)	9 / 33(0)	9 / 40(0)	9 / 40(0)	10 / 90(0)	9 / 69(0)	9 / 11(0)	9 / 11(0)	2 / 1(0)	9 / 9(0)	10 / 27(0)
	AUPRC	0.53	0.63	0.61	0.64	0.68	0.71		0.78	0.78	0.78	0.81	0.68
Scenario 6a	Selected biomarkers	2	25	15	29	37	109	99	23	14	1	13	38
	TP / FP (pFP)	1 / 1(0)	4 / 21(1)	4 / 11(0)	6 / 23(1)	7 / 30(1)	10 / 99(10)	9 / 90(0)	9 / 14(0)	8 / 7(0)	1 / 0(0)	8 / 5(0)	10 / 29(1)
	AUPRC	0.27	0.25	0.37	0.38	0.43	0.21		0.75	0.69	0.71	0.71	0.62
Scenario 4b	Selected biomarkers	4	13	13	14	14	25	18	2	2	0	2	7
	TP / FP (pFP)	1 / 3(0)	1 / 12(0)	1 / 12(0)	1 / 13(0)	1 / 13(0)	1 / 24(0)	1 / 17(0)	1 / 1(0)	1 / 1(0)	0 / 0(0)	1 / 1(0)	1 / 6(0)
	AUPRC	1.00	0.99	0.98	0.94	0.98	1.00		0.98	0.99	0.98	0.99	0.99
Scenario 5b	Selected biomarkers	19	55	50	64	62	127	101	26	31	6	26	46
	TP / FP (pFP)	8 / 11(0)	14 / 41(0)	14 / 36(0)	16 / 48(0)	16 / 46(0)	20 / 107(0)	18 / 83(0)	13 / 13(0)	15 / 16(0)	4 / 2(0)	15 / 12(0)	16 / 31(0)
	AUPRC	0.42	0.45	0.45	0.49	0.51	0.53		0.63	0.63	0.62	0.65	0.51
Scenario 6b	Selected biomarkers	1	20	12	30	39	124	110	28	14	1	13	35
	TP / FP (pFP)	1 / 1(0)	4 / 16(1)	4 / 8(0)	7 / 22(1)	9 / 30(1)	19 / 106(20)	17 / 93(1)	13 / 15(1)	8 / 6(0)	0 / 0(0)	8 / 6(0)	13 / 22(1)
	AUPRC	0.19	0.16	0.26	0.27	0.28	0.17		0.54	0.48	0.47	0.48	0.35
Scenario 4c	Selected biomarkers	1	8	8	6	8	12	14	0	1	0	2	4
	TP / FP (pFP)	0 / 0(0)	0 / 8(0)	0 / 8(0)	0 / 6(0)	0 / 7(0)	1 / 12(0)	1 / 13(0)	0 / 0(0)	0 / 1(0)	0 / 0(0)	0 / 2(0)	0 / 4(0)
	AUPRC	0.36	0.34	0.34	0.25	0.34	0.44		0.33	0.35	0.25	0.37	0.40
Scenario 5c	Selected biomarkers	3	28	27	23	32	51	51	4	9	0	8	21
	TP / FP (pFP)	1 / 1(0)	5 / 23(0)	5 / 22(0)	4 / 19(0)	5 / 27(0)	7 / 44(0)	7 / 44(0)	2 / 2(0)	4 / 6(0)	0 / 0(0)	3 / 5(0)	5 / 16(0)
	AUPRC	0.27	0.29	0.32	0.27	0.33	0.44		0.33	0.35	0.27	0.35	0.35
Scenario 6c	Selected biomarkers	2	22	17	19	27	73	75	6	8	0	7	27
	TP / FP (pFP)	1 / 1(0)	3 / 19(0)	3 / 14(0)	3 / 16(0)	4 / 22(1)	7 / 66(8)	7 / 68(3)	3 / 4(0)	3 / 5(0)	0 / 0(0)	3 / 4(0)	5 / 22(0)
	AUPRC	0.21	0.22	0.26	0.24	0.29	0.19		0.32	0.33	0.26	0.34	0.31

TP: true positive, FP: false positive, pFP: prognostic false positive, AUPRC: area under the precision‐recall curve.

The **univariate approach** controlled well the type‐I error in null scenarios (Table 2), but was very conservative in alternative scenarios, especially in presence of main effects (Table 3). This is illustrated by low FDR and large FNR (Fig. 1), and by very low power (e.g., 0.59 and 0.43 in scenarios 6a and 6b, Table 2). Low FDR and large FNR are not due to the adjusted *p* threshold chosen for discarding biomarkers, as shown by the AUPRC, which is often ≤0.5 (Table 3). Consequently, the interaction strength is low (Fig. 2).

The second group contains methods not performing selection of the main effects: the **modified covariates**, dimension reduction approaches (**PCA+lasso** and **PLS+lasso**), and **ridge+lasso**. In the null scenarios, their type‐I error was moderate (0.24‒0.58) to high (0.49‒0.61) with low and high censoring, respectively (Table 2). In alternative scenarios (Fig. 1, Table 3) with no main effects (scenarios 4–5), these methods identified most of the treatment‐effect modifiers (small FNR), but together with many FPs (large FDR); they predicted fairly well the individual treatment benefit (Fig. 2). However, in presence of main effects (scenario 6), these methods were more conservative and missed most of treatment‐effect modifiers, implying low power (Table 2) and low interaction strength (Fig. 2); these figures were lower for *p* = 1000 than for *p* = 500. In general, the PLS+lasso and ridge+lasso, which account for the outcome in dimension reduction of the main effect matrix, performed slightly better than the PCA+lasso and modified covariates. This was less evident with smaller biomarker effects (scenario 6c). In addition, the poor results of the modified covariates were amplified when treatment‐effect modifiers were also correlated with prognostic biomarkers (Supporting Information Fig. S2) while the PCA+lasso and the PLS+lasso were not impacted by such correlation. The AUPRC of these four methods was often ≤0.5. Of note, in the alternative scenarios, their interaction strength ΔC‐statistics were highly impacted by overfitting (Fig. 2), especially for the modified covariates and the ridge+lasso.

In the third group, the **group‐lasso** and the **two‐I model** identified properly the treatment‐effect modifiers in general, but often together with several prognostic biomarkers, notably in null scenarios. In null scenarios without prognostic biomarkers (1–2a and 1–2b), the two‐I model and the group‐lasso had type‐I error of 0.38–0.44 and 0.48–0.56, respectively, which increased to 1 in presence of main effects (scenario 3, Table 2). On the other hand, these methods identified almost all the treatment‐effect modifiers in alternative scenarios (Table 3), but with a lot (often >100) of FPs, notably for *p* = 1000. This led to very high FDR (Fig. 1) and power (Table 2). Despite the high number of FPs, the group‐lasso had fairly large AUPRC (Table 3). However, in the scenario 6, it selected all the prognostic biomarkers (i.e., FPp), thus yielding high FDR and low AUPRC. Both the two‐I model and the group‐lasso had quite high ΔC‐statistics (Fig. 2).

In the fourth group, we found methods that do not impose the hierarchy constraint: the **full‐lasso** and the three kinds of **adaptive lasso** (with either specific (Sw), grouped (Gw), or arm‐specific prognostic (Aspw) weights). In the null scenarios 1 and 2, the adaptive lasso (Sw or Gw) and the full‐lasso selected no biomarkers in most of cases, resulting in a low type‐I error (Table 2). However, the latter was highly affected by the prognostic biomarkers (scenarios 3a and 3b, type‐I error: 0.88–0.98). The adaptive lasso (Aspw) had a moderately high type‐I error in scenarios 1–2a and 1–2b (0.36–0.42), and large in presence of only prognostic biomarkers (0.55, *p* = 1000). In the alternative scenarios, the full‐lasso and two adaptive lassos (Sw and Aspw) identified most of the treatment‐effect modifiers, with slightly worse results for *p* = 1000 than for *p* = 500. With *p* = 500, they well identified the treatment‐effect modifiers with few FPs and performed the best in terms of FNR, FDR (Table 3, Fig. 1) and interaction strength (Fig. 2). However, with *p* = 1000, the adaptive lasso (Sw and Aspw) performed much worse in presence of main effects (scenario 6b) by selecting much less biomarkers (on average: 14 and 13 biomarkers, respectively, and FNR = 0.61, Table 3); this poor results were amplified when treatment‐effect modifiers were also correlated with prognostic biomarkers (Supporting Information Fig. S2). The adaptive lasso (Gw) selected the null model in most of cases (leading to a FDR ≈ 0, FNR ≈ 1, low power and interaction strength; Table 2, Figs. 1 and 2). However, in terms of AUPRC, it performed as well as the full‐lasso and the two other versions of adaptive lasso (Table 3). This means that the selected tuning parameter is too high and that the method could perform better by selecting a lower parameter. With higher censoring and lower effects sizes (scenarios 4–6c), the four methods performed similarly to the other methods, by selecting fewer biomarkers (Table 3) and with low AUPRC (Table 3). Although not forced, the hierarchy constraint is maintained for more than 50% of the interactions in alternative scenarios.

The **gradient boosting** did not behave like any of the abovementioned groups of methods. In null scenarios, similarly to the group‐lasso, it had high type‐I error (0.66–1) even with low censoring (scenarios 1–3a and 1–3b; Table 2). However, in alternative scenarios, it performed as well as the methods not imposing the hierarchy constraint: good selection of the treatment‐effect modifiers, with few FPs (Table 3). In addition, it was not that conservative with *p* = 1000. The gradient boosting had quite good interaction strength, but was highly impacted by overfitting in some scenarios (Fig. 2).

In the sensitivity analysis generating data from Weibull distribution, the variation of the hazards over time (decreasing or increasing) did not affect the relative performances of any method (Supporting Information Fig. S1).

## 4 Application

We applied the methods compared so far to publicly available data from the Gene Expression Omnibus database (http://www.ncbi.nlm.nih.gov/geo, last accessed on June 1, 2016) to identify treatment‐effect modifiers in 614 breast cancer patients (Desmedt et al., 2011; Hatzis et al., 2011) receiving anthracycline‐based adjuvant chemotherapy with (*n* = 507) or without (*n* = 107) taxane. The three‐year distant‐relapse free survival was 78% (95% CI: 74–82%) and 79% (95% CI: 71–87%), respectively. We preprocessed the expression data of 22,277 genes (Affymetrix array) via frozen robust multiarray (McCall et al., 2010) and cross‐platform normalization (Shabalin et al., 2008). We removed genes with interquartile range ≤1. We standardized the remaining *p* = 1689 genes.

Patients were randomly assigned to a training set (315 patients) and a validation set (299 patients). The results (Table 4) show a large spread in the number of selected biomarkers between methods: 0–39. The modified covariates, PCA+lasso, PLS+lasso, and ridge+lasso were highly concordant, with 14 biomarkers selected by at least three methods. Most of those selected by the two‐I model (10/34) and group lasso (3/4) were not selected by the other methods. All the biomarkers selected by the univariate approach were also selected by the gradient boosting. The alasso (Aspw) identified two genes and the alasso (Sw) identified only the IFIH1 gene, common to almost all the other methods except for the group‐lasso and the univariate approaches. Importantly, the IFIH1 gene expression is already known for being associated with recurrence in nonresponders to taxane‐based chemotherapy in early breast cancer (Magbanua et al., 2015) and is included in two patents to predict the benefit of taxanes (Gehrmann and Von Törne, 2009; Wang et al., 2014). Functional studies also suggested that the IFIH1 expression is associated with resistance to taxanes in prostate cancer (Marín‐Aguilera et al., 2011). This single gene had a moderate interaction strength (ΔC‐statistics = 0.06). By selecting more biomarkers, the other methods slightly improved interaction strength (Table 4). Interestingly, the gradient boosting and the alasso (Aspw) had the highest ΔC‐statistics (0.18 and 0.14, respectively).

**Table 4:** Selected treatment‐effect modifiers and interaction strength of the developed signature in the breast cancer application

	Number of selected biomarkers	ΔC‐statistics
**Univariate**	4	0.10
**Modified covariates**	21	0.09
**PCA+lasso**	13	0.12
**PLS+lasso**	20	0.01
**Ridge+lasso**	39	0.04
**Group‐lasso**	4	0.06
**Two‐I model**	34	0.12
**Full‐lasso**	0	0
**Alasso (Sw)**	1	0.06
**Alasso (Gw)**	0	0
**Alasso (Aspw)**	2	0.14
**Gradient boosting**	8	0.18

## 5 Discussion

Parsimonious gene signatures aim at selecting patients more likely to benefit from a treatment (Buyse and Michiels, 2013; Hingorani et al., 2013). Although this needs identifying the treatment‐effect modifiers (Michiels et al., 2011), no clear guidance has been established yet on how to do in high‐dimensional spaces. Biomarker selection can have multiple aims, at least two of which of particular interest: selecting markers which have a biologic role and selecting patients likely to benefit from the therapy. In this paper, we focused on sparse selection and compared 12 methods to find treatment‐effect modifiers while limiting the FP selection. Indeed, for practical use of the biomarker score, determining a few biomarkers with high accuracy (e.g., ELISA) is usually preferred compared to less‐accurate technologies (e.g., mass spectroscopy). Obviously, the chosen methods undersample all the possible approaches. We also proposed a novel metric about interaction strength prediction because this is tightly related to correct biomarker selection.

Based on the results of the simulation study, different groups of methods were identified. First, the straightforward way for identifying interactions via univariate models should be avoided when biomarkers are correlated, even with FDR control. If a method starting from the null model would be considered, the gradient boosting is an option, but it does not control at all the type‐I error in null scenarios. Methods that do not perform selection of the main effects are quite well powered, except in presence of prognostic biomarkers as compared to methods performing selection on the main effects. Of note, keeping all the main effects could be a practical drawback, as sparse prognostic signatures can be assessed easily and reliably on different platforms (e.g., by RT‐PCR). Dimension reduction of the main effect matrix independently of the outcome (PCA) has negative impact in presence of strong main effects. Conversely, methods performing variable selection on the main effects perform globally well, irrespective of whether the hierarchy constraint is forced. As an exception, the group‐lasso is highly impacted by the presence of main effects due to the groups that can not separate the main effect and the interaction of each biomarker. To overcome this drawback, testing strategies could be considered to evaluate remaining interactions (Lockhart et al., 2014). In any case, further arbitrary choices would be required. Another further development could consist in an adaptive group‐lasso with weights based on the interaction only. The model estimating the arm‐specific prognostic effects identified rather well the treatment‐effect modifiers in the simulations study. Nevertheless, to allow for treatment modifiers that have prognostic effects in each of the treatment arms, a contrast test would be needed between the two prognostic effects, which is not straightforward in a penalized model. Finally, we would like to stress: (i) the importance of external data for investigating the interaction strength, as most of the methods are impacted by overfitting, notably the modified covariates and the two‐step methods; (ii) and that the identification of interactions is difficult and requires a lot of events, whatever the method.

For all but one methods, the list of interactions retained is defined by a tuning parameter estimated via the Verweij and van Houwelingen (1993, 1994) CV criterion. However, note that this technique is sometimes suboptimal, finding too conservative tuning parameters for the adaptive lasso (Gw) and too lenient ones for the group‐lasso. For this latter case, we previously reviewed and compared empirical extensions of the lasso penalty to reduce the FPs in high‐dimensional Cox regression models (Ternès et al., 2016).

In the application to the gene expression data set in breast cancer, the methods behaved quite differently in terms of number of selected biomarkers and of interaction strength. This application can only be viewed as an illustration, as no gold standard is available to infer which biomarker truly modifies the treatment effect and the expression data were not collected in the context of a randomized clinical trial. However, the gene selected by most of the methods was already suggested to predict resistance to taxane‐based chemotherapy in the literature. Adjusting the models on clinical factors was beyond the aim of the present study but is another interesting point. Such factors could be considered as prognostic factors not subjected to competitive selection, or they could be used as candidates for treatment‐covariate interactions, just like the biomarkers. Yet, no consensus has been reached on how to deal with clinical factors in this context. For a fair comparison, we estimated the effect of the clinical factors (nodal status and tumor grade) and fixed them as an offset for all the methods. The R code used to download the clinical and gene expression data and to apply the 12 methods is available as supplementary material.

## Conflict of interest

*The authors have declared no conflict of interest*.

## Supporting information

Figure S1: False negative rate against the false discovery rate in alternative scenarios for time‐decreasing
(1st row) and time‐increasing (2nd row) hazards. Average quantities across 250 replications.

Figure S2: False negative rate against the false discovery rate in alternative scenarios for several
correlation structures between active markers. Average quantities across 250 replications.

Supporting FigureS1

Supporting Information
