# Supplementary material for: Identification of biomarker‐by‐treatment interactions in randomized clinical trials with survival outcomes and high‐dimensional spaces
Source: Biom J. 2016 Nov 15;59(4):685–701. doi: 10.1002/bimj.201500234 (PMC5763402; doi:10.1002/bimj.201500234)
Supplement: Supplementary file 1 — Figure S1: False negative rate against the false discovery rate in alternative scenarios for time‐decreasing (1st row) and time‐increasing (2nd row) hazards. Average quantities across 250 replications. [file BIMJ-59-685-s001.pdf]

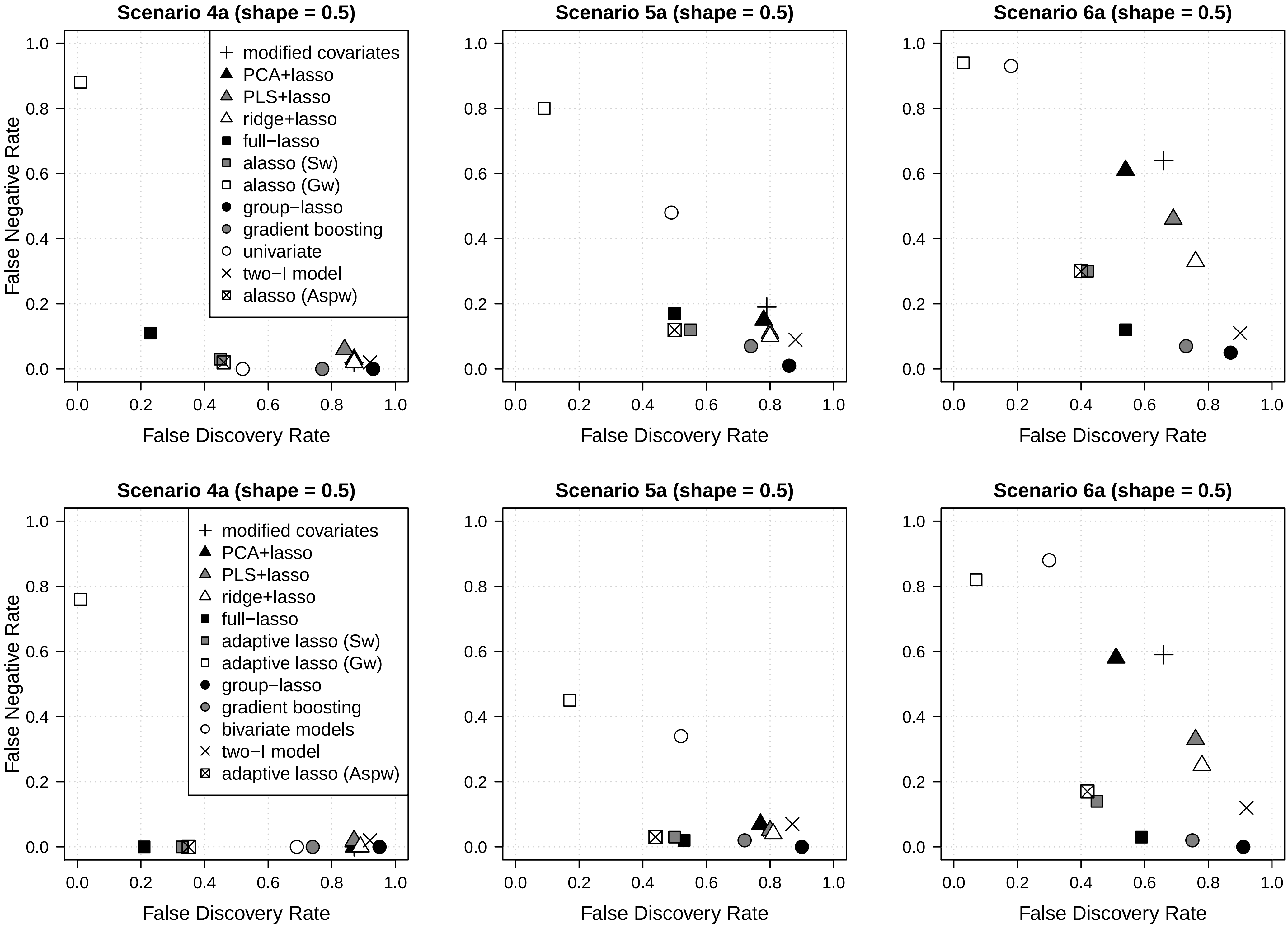
Scenario 4a (shape = 0.5)
Scenario 5a (shape = 0.5)
Scenario 6a (shape = 0.5)
False Negative Rate
False Discovery Rate
modified covariates
PCA+lasso
PLS+lasso
ridge+lasso
full−lasso
alasso (Sw)
alasso (Gw)
group−lasso
gradient boosting
univariate
two−I model
alasso (Aspw)
Scenario 4a (shape = 0.5)
Scenario 5a (shape = 0.5)
Scenario 6a (shape = 0.5)
modified covariates
PCA+lasso
PLS+lasso
ridge+lasso
full−lasso
adaptive lasso (Sw)
adaptive lasso (Gw)
group−lasso
gradient boosting
bivariate models
two−I model
adaptive lasso (Aspw)
