# Supplementary material for: Identification of biomarker‐by‐treatment interactions in randomized clinical trials with survival outcomes and high‐dimensional spaces
Source: Biom J. 2016 Nov 15;59(4):685–701. doi: 10.1002/bimj.201500234 (PMC5763402; doi:10.1002/bimj.201500234)
Supplement: Supplementary file 2 — Figure S2: False negative rate against the false discovery rate in alternative scenarios for several correlation structures between active markers. Average quantities across 250 replications. [file BIMJ-59-685-s002.pdf]

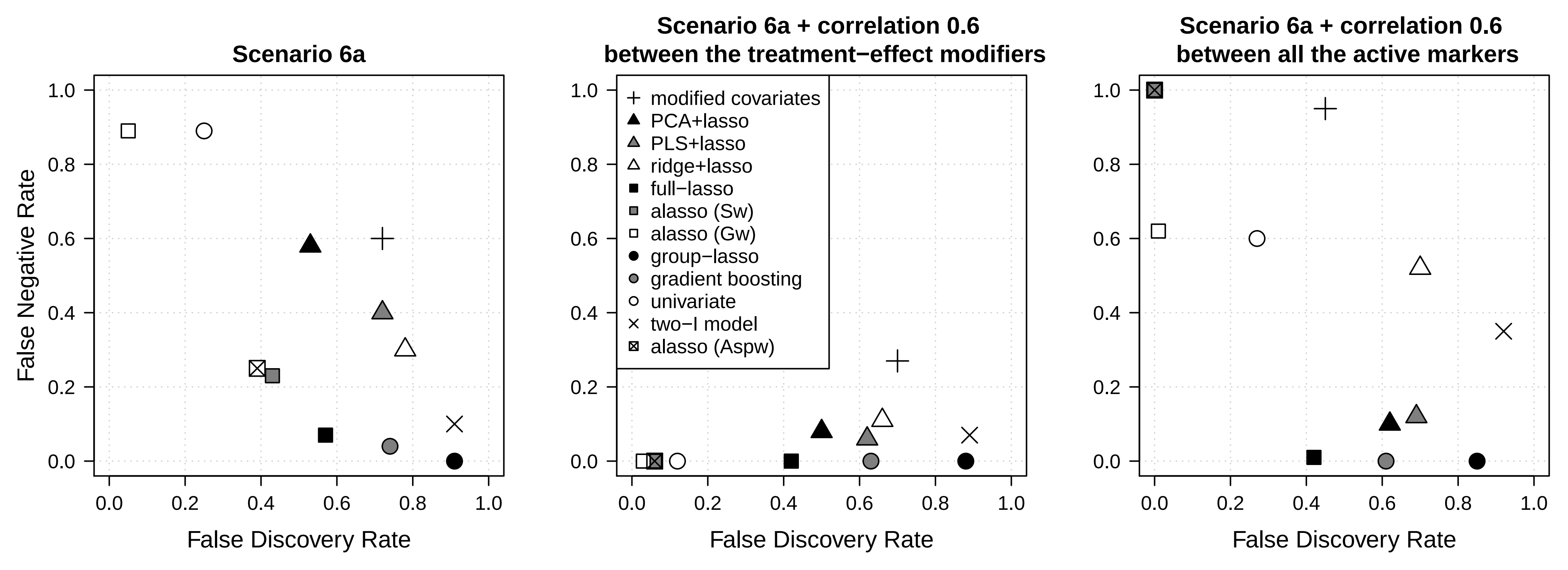

Scenario 6a
Scenario 6a + correlation 0.6 between the treatment−effect modifiers
Scenario 6a + correlation 0.6 between all the active markers
False Negative Rate
False Discovery Rate
0.0
0.2
0.4
0.6
0.8
1.0
modified covariates
PCA+lasso
PLS+lasso
ridge+lasso
full−lasso
alasso (Sw)
alasso (Gw)
group−lasso
gradient boosting
univariate
two−I model
alasso (Aspw)
