# Supplementary material for: Identification of biomarker‐by‐treatment interactions in randomized clinical trials with survival outcomes and high‐dimensional spaces
Source: Biom J. 2016 Nov 15;59(4):685–701. doi: 10.1002/bimj.201500234 (PMC5763402; doi:10.1002/bimj.201500234)

Supplementary Figure legends

SUPPLEMENTARY FIGURE 1:

TITLE: False Negative Rate against the False Discovery Rate in alternative scenarios for time-decreasing (1st row) and time-increasing (2nd row) hazards

LEGENDS: Average quantities across 250 replications.

SUPPLEMENTARY FIGURE 2:

TITLE: False Negative Rate against the False Discovery Rate in alternative scenarios for several correlation structures between active markers

LEGENDS: Average quantities across 250 replications.

Supplementary Figure 1

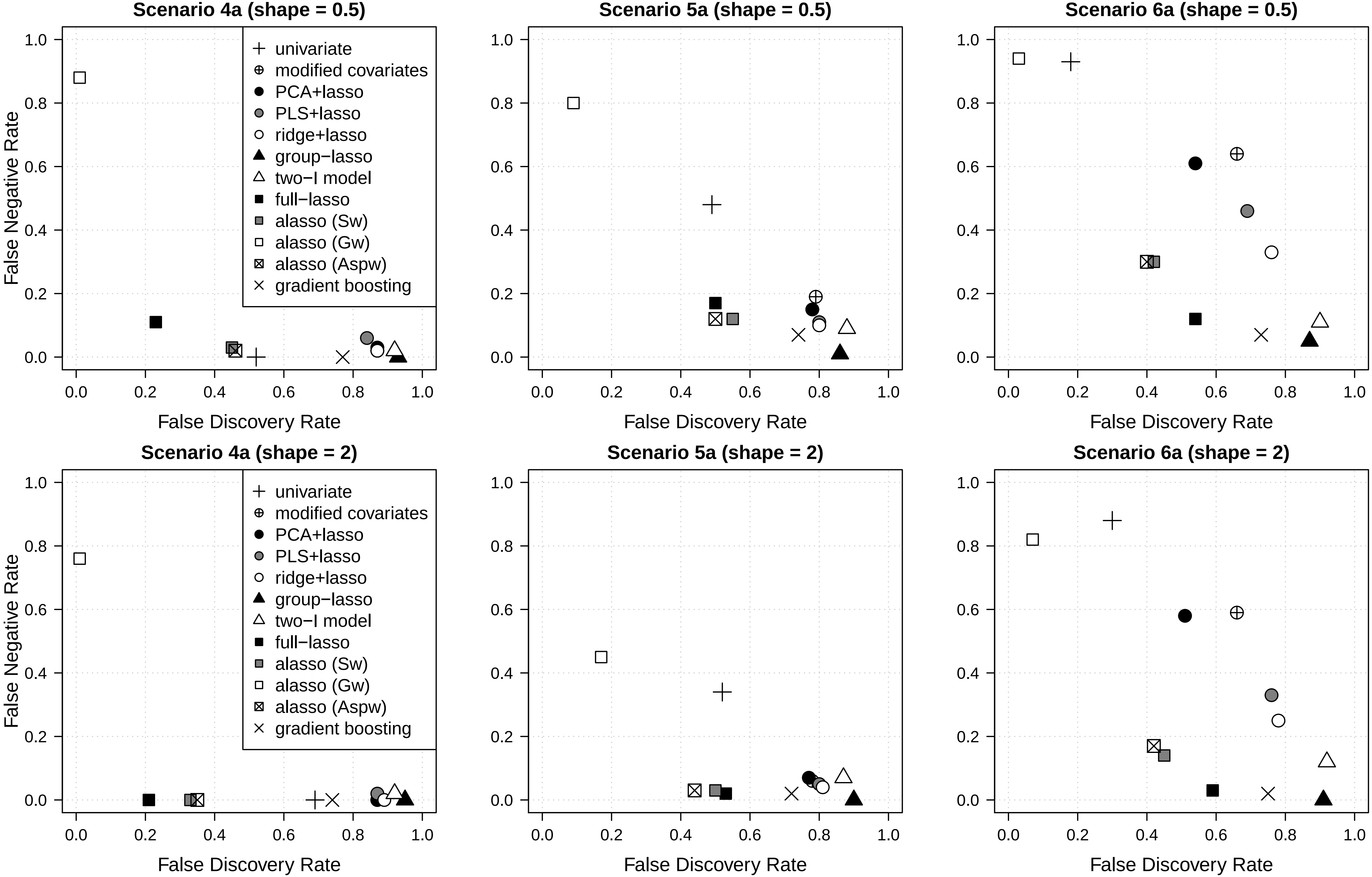

Supplementary Figure 2

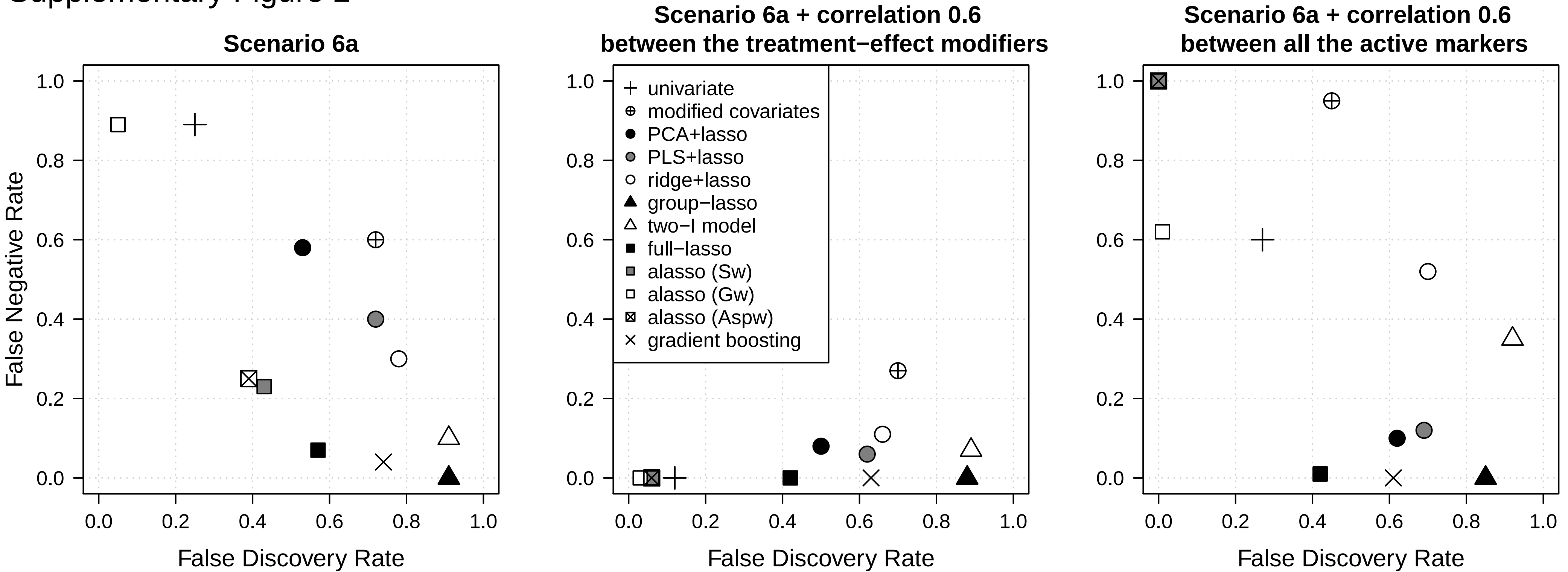

Supplement: Supplementary file 3 — Supporting FigureS1 [file BIMJ-59-685-s003.pdf]
