# Supplementary material for: Identification of biomarker‐by‐treatment interactions in randomized clinical trials with survival outcomes and high‐dimensional spaces
Source: Biom J. 2016 Nov 15;59(4):685–701. doi: 10.1002/bimj.201500234 (PMC5763402; doi:10.1002/bimj.201500234)
Supplement: Supplementary file 4 — Supporting Information [file BIMJ-59-685-s004.zip › Code_and_Data/case_study/data/README_Breast.pdf]

Example: Breast data

The data are publicly available from the Gene Expression Omnibus database (GSE 16446 and 25066). For more information of the data, see Desmedt et al. (JCO, 2011) and Hatzis et al. (JAMA, 2011).

The data concerns n = 614 early breast cancer patients treated by anthracycline only (A) or anthracycline + taxane-based (AT) chemotherapy.

The initial data set contained continuous expression of 22,277 genes (Affymetrix array). To make microarrays comparable, we preliminarily normalized expression data using the frozen RMA (McCall et al., 2004) and XPN (Shabalin et al., 2008) technics.

Then, we reduced the number of analysed genes by filtering out those with interquartile range less or equal to 1 (Gentleman et al., 2006).

Finally, the Breast data set contains p = 1689 normalized gene expression variables, plus three further variables: the treatment arm (*treat*, coded as +0.5 for AT and -0.5 for A) and the distant recurrence free survival (DRFS) outcome (*time* in years and *status*, coded as 0 for censored and 1 for event).

References:

Desmedt, C., Di Leo, A., de Azambuja, E., Larsimont, D., Haibe-Kains, B., Selleslags, J., Delaloge, S., Duhem, C., Kains, J.-P., Carly, B., Maerevoet, M., Vindevoghel, A., Rouas, G., Lallemand, F., Durbecq, V., Cardoso, F., Salgado, R., Rovere, R., Bontempi, G., Michiels, S., Buyse, M., Nogaret, J.-M., Qi, Y., Symmans, F., Pusztai, L., D'Hondt, V., Piccart-Gebhart, M. and Sotiriou, C. (2011). Multifactorial approach to predicting resistance to anthracyclines. *Journal of Clinical Oncology* **29**, 1578–1586.

Gentleman, R., Carey, V., Huber, W., Irizarry, R., & Dudoit, S. (2006). Bioinformatics and computational biology solutions using R and Bioconductor. Springer Science & Business Media.

Hatzis, C., Pusztai, L., Valero, V., Booser, D. J., Esserman, L., Lluch, A., Vidaurre, T., Holmes, F., Souchon, E., Wang, H., Martin, M., Cotrina, J., Gomez, H., Hubbard, R., Chacón, J. I., Ferrer-Lozano, J., Dyer, R., Buxton, M., Gong, Y., Wu, Y., Ibrahim, N., Andreopoulou, E., Ueno, N. T., Hunt, K., Yang, W., Nazario, A., DeMichiele, A., O'Shaughnessy, J., Hortobagyi, G. N. and Symmans, W. F. (2011). A genomic predictor of response and survival following taxane-anthracycline chemotherapy for invasive breast cancer. *Journal of the American Medical Association* **305**, 1873–1881.

McCall, M. N., Bolstad, B. M. and Irizarry, R. A. (2010). Frozen robust multiarray analysis (fRMA). *Biostatistics* **11**, 242–253.

Shabalin, A. A., Tjelmeland, H., Fan, C., Perou, C. M. and Nobel, A. B. (2008). Merging two gene-expression studies via cross-platform normalization. *Bioinformatics* **24**, 1154–1160.
